# Supplementary material for: Homeostatic reinforcement learning for integrating reward collection and physiological stability
Source: eLife. 2014 Dec 2;3:e04811. doi: 10.7554/eLife.04811 (PMC4270100; doi:10.7554/eLife.04811)
Supplement: Figure 5—source data 1. — DOI: http://dx.doi.org/10.7554/eLife.04811.009 [file elife04811s002.docx]

| Parameter | Value | Explanation |
| --- | --- | --- |
| $\alpha$ | 0.25 | Learning rate |
| $\beta$ | 10 | Exploration rate in the soft-max rule |
| $\gamma$ | 0.8 | Discount factor |
| $m$ | 2 | Free parameter of the drive function |
| $n$ | 4 | Free parameter of the drive function |
| $H^{*}$ | 0 | Homeostatic setpoint |
| $-$ | 0 | Initial internal state |
