# Supplementary material for: Homeostatic reinforcement learning for integrating reward collection and physiological stability
Source: eLife. 2014 Dec 2;3:e04811. doi: 10.7554/eLife.04811 (PMC4270100; doi:10.7554/eLife.04811)
Supplement: Figure 12—source data 1. — DOI: http://dx.doi.org/10.7554/eLife.04811.029 [file elife04811s006.docx]

| Parameter | Value | Explanation |
| --- | --- | --- |
| $\alpha_{1}$ | 0.4 | Learning rate for outcome functions |
| $\alpha_{2}$ | 0.2 | Learning rate for transition functions |
| $\beta$ | 0.25 | Exploration rate in the soft-max rule |
| $\gamma$ | 1 | Discount factor |
| $m$ | 3 | Free parameter of the drive function |
| $n$ | 4 | Free parameter of the drive function |
| $H^{*}$ | 200 | Homeostatic setpoint |
| $-$ | 0 | Initial internal state |
| $-$ | 50 | Effect of 1g of outcome on the homeostatic variable |
| $-$ | ~3 | Energy cost for every action |
